# SARS-CoV-2 Prediction Strategy Based on Classification Algorithms from a Full Blood Examination

**DOI:** 10.1155/2023/3248192

**Published:** 2023-08-22

**Authors:** C. F. Choukhan, I. Lasri, R. El Hatimi, M. R. Lemnaouar, M. Esghir

**Affiliations:** ^1^Laboratory of Mathematics, Computing and Applications, Mohammed V University in Rabat, Faculty of Sciences, Rabat, Morocco; ^2^Laboratory of Conception and Systems (Electronics, Signals and Informatics), Mohammed V University in Rabat, Faculty of Sciences, Rabat, Morocco; ^3^LASTIMI, Mohammed V University in Rabat, Superior School of Technology, Sale, Rabat, Morocco

## Abstract

A fast and efficient diagnosis of serious infectious diseases, such as the recent SARS-CoV-2, is necessary in order to curb both the spread of existing variants and the emergence of new ones. In this regard and recognizing the shortcomings of the reverse transcription-polymerase chain reaction (RT-PCR) and rapid diagnostic test (RDT), strategic planning in the public health system is required. In particular, helping researchers develop a more accurate diagnosis means to distinguish patients with symptoms with COVID-19 from other common infections is what is needed. The aim of this study was to train and optimize the support vector machine (SVM) and K-nearest neighbors (KNN) classifiers to rapidly identify SARS-CoV-2 (positive/negative) patients through a simple complete blood test without any prior knowledge of the patient's health state or symptoms. After applying both models to a sample of patients at Israelita Albert Einstein at São Paulo, Brazil (solely for two examined groups of patients' data: “regular ward” and “not admitted to the hospital”), it was found that both provided early and accurate detection, based only on a selected blood profile via the statistical test of dependence (ANOVA test). The best performance was achieved by the improved SVM technique on nonhospitalized patients, with precision, recall, accuracy, and AUC values reaching 94%, 96%, 95%, and 99%, respectively, which supports the potential of this innovative strategy to significantly improve initial screening.

## 1. Introduction

The World Health Organization designated SARS-CoV-2 (COVID-19) as a pandemic on March 11, 2020 [1]. The rapid propagation of the disease around the world has increased the need to apply health protection measures. These measures were aimed at solving the problem of overburdened intensive care units, as well as to strengthen and preserve the capacity of hospitals. As a result, many countries have adopted new health approaches and diverse perspectives to prevent the excess spread of the virus in terms of virus vitality within a specific political-economic territory. Examples include the closure of borders and the cancellation of sporting and cultural events. Unfortunately, these decisions have caused economic, social, and environmental disruptions. In addition, they have brought uncertainties and fears to the world economy, education, health, and the fundamental rights of the population.

As of September 30, 2022 [2], more than 622,585,710 cumulative cases of SARS-CoV-2 have been confirmed worldwide, along with more than 6,547,814 deaths in 228 countries and territories. Approximately 40% of cases present with mild disease (cough and fever), 40% with moderate disease (bilateral pneumonia), 15% with severe disease, and 5% with critical disease [3]. The severe consequences of COVID-19 are due to its rapid spread, the inability to make a quick and accurate diagnosis, and the inability to perform large-scale testing of patients. It is therefore crucial to establish rapid and reliable diagnostic methods to detect the disease in real time [4].

Indeed, healthcare is a vast sector that requires the collection, analysis, and processing of medical data, which have recently become impossible due to several factors, such as massive data volumes, the inadequacy of wireless network applications, and security issues [5]. Hence, it is essential to use data mining to find and extract rich information for classification. Medical datasets can be used to precisely detect SARS-CoV-2 infections [6]. However, the primary limiting factor is data processing, which necessitates real-time data collection and the provision of data to researchers for immediate medical response.

In the same vein, artificial intelligence (AI) promises to transform the healthcare sector [7]. Machine learning (ML) and deep-learning algorithms are capable of detecting COVID-19 [8]. In fact, the classification is one process by which COVID-19 patients are assigned to their corresponding classes [9]. There are many classification methods, such as the Bayesian method, AdaBoost, random forest, artificial neural networks, and K-nearest neighbors [9].

Much COVID-19 research has focused on how AI can be deployed to detect, confirm, and make forecasts at early stages. As the authors in [10] involved regression models (CUBIST, RF, RIDGE, SVR, and stacked-set learning), the ARIMA statistical model has also been used in some cases to make similar predictions. The authors in [11] adopted BRNN, KNN, QRF, and SVR as well as the VDM approach coupled with exogenous climate variables to predict confirmed cumulative cases in ten Brazilian states. These predictions were made one, three, and six days in advance. As reported in [12], the SVM model detected and discriminated patients with severe COVID-19 from those with mild symptoms using 28 features based on clinical information and blood/urine test data, with an overall accuracy of 0.8148.

Previously, an efficient scheme in [13] was proposed using the available, relevant X-ray images to train an efficient deep neural AI network and use the trained parameters to detect COVID-19 cases even with a very small sample of COVID-19 X-rays. The proposed method provided a very satisfactory detection performance at 97.4% accuracy.

A case report in [14] emphasized the importance of full autopsy in understanding the disease process and identifying potential targets for therapeutic interventions. The authors of the aforementioned study conducted a full autopsy on a confirmed COVID-19 patient in Lagos, Nigeria, providing valuable insights into the pathological features of the disease.

Two studies proposed different machine-learning approaches for addressing COVID-19 challenges. Ribeiro et al. [15] proposed the use of ensemble-learning models coupled with urban mobility information to predict COVID-19 incidence cases. This approach leverages the relationship between human mobility patterns and the spread of the virus to achieve accurate predictions. Another study [16] introduced an equilibrium-based COVID-19 diagnostic method using routine blood tests and a sparse deep convolutional model. This method provides a noninvasive, low-cost, and potentially more accurate alternative to existing diagnostic methods.

Da Silva et al. [11] focused on using climatic exogenous variables to forecast COVID-19 cases. This study proposed a novel approach for forecasting Brazilian and American COVID-19 cases based on artificial intelligence coupled with climatic exogenous variables, providing a more holistic approach to COVID-19 prediction.

In [17], the researchers applied AI to identify commercially available medicines that may be effective in treating patients with COVID-19. At the core of their proposed model, they implemented the bidirectional encoder representations from transformers (BERTs) framework.

COVID-19 primarily affects the respiratory system. Thus, in [18], the authors presented a fine-tuned model based on a generative adversarial network to detect one of the symptoms of COVID-19 infection from chest X-ray scans. Gunraj et al. [19] applied a convolutional neural network model to detect COVID-19 in patients using chest X-ray images. They used pretrained ImageNet and trained the model on an open-source dataset of X-ray images. Aggarwal et al. [20] reviewed and summarized a number of important research papers on deep learning-based classification of COVID-19 across CXR and CT images. Using a deep learning-based P-shot N-ways Siamese network as well as prototypical nearest neighbor classifiers, classification of COVID-19 infection from lung CT slices was proposed by the authors in [21]. Another approach for classification of COVID-19 chest X-ray images from two different datasets (small and large datasets) using a tunable Q-wave transform (TQWT) based on a memristive crossbar array (MCA) was proposed by the authors in [22]. The average accuracy values obtained for the proposed method are 98.82% and 94.64%, respectively.

Together, these studies highlight the potential of different approaches; therefore, it is necessary to construct prediction techniques and innovative applications for frequent diseases, as well as to further expand prediction methodologies. The objective of the present study is to address this need. The main contributions of the proposed work that have not been addressed in the prior art are as follows:

- We showed that it is possible to predict whether a person is positive or negative for COVID-19 infection in the early stage of the disease, using anonymized data from Israelita Albert Einstein Hospital [23]. The data analysis process consists of two stages: statistical analysis followed by data processing with machine-learning algorithms using SVM and KNN. The enhanced SVM technique achieved high values for precision, recall, accuracy, and AUC, with scores reaching 94%, 96%, 95%, and 99%, respectively.
- Without knowledge of any individual's medical history or symptoms, the proposed method for predicting the COVID-19 test result (positive/negative) is solely based on a complete blood examination.
- This strategy is based on the need to rapidly distinguish patients with COVID-19 from those with similar symptoms, as well as the recognized limitations of using the RT-PCR and rapid diagnostic test (RDT).
- The statistical test and feature selection technique plays a crucial role in the prediction model.
- Polynomial features and SelectKbest provide more information about the most important variables.
- This study indicates the immediate relation of the pathogenesis of COVID-19 to monocytes and neutrophils, as shown by the results of the dependence test (ANOVA test); it should be noted that the four variables from the regular ward data had the best scores for eosinophils, followed by red blood cells, hemoglobin, and leukocytes. Community patients with SARS-CoV-2 have high scores on leukocyte, monocyte, platelet, and eosinophil parameters.

This article is structured as follows: Section 2 includes a description of the dataset, the data analysis and data preprocessing for the classification algorithms used, and a detailed description of the materials and methodology. The results of the experiment are presented in Section 3.  Section 4 discusses the results, and Section 5 offers conclusions on the prospects for use of this analysis procedure to detect COVID-19.

## 2. Materials and Methods

### 2.1. Dataset

The data used in this study were obtained from the Kaggle website [23]. Information was retrieved for patients treated at Israelita Albert Einstein Hospital in São Paulo, Brazil, who had samples collected to perform the SARS-CoV-2 RT-PCR and additional laboratory tests between March 28, 2020, and April 3, 2020 [24]. Following international best practices, all data were anonymized. The normalization process resulted in a mean of 0 and a standard deviation value for all clinical data.

The hospital data consisted of 5,644 individual patients and 111 variables, as presented in Table 1. The patients were classified into four groups: community (not admitted to hospital), regular ward, semi-intensive unit, and intensive care unit (see Table 2).

Patient information included age, the SARS-CoV-2 RT-PCR test result, and full blood results, including hematocrit, hemoglobin, platelets, mean platelet volume, red blood cells, lymphocytes, mean corpuscular hemoglobin concentration (MCHC), leukocytes, basophils, mean corpuscular hemoglobin (MCH), eosinophils, mean corpuscular volume (MCV), monocytes, and red blood cell distribution width (RDW). Additional pathogen tests were conducted on 356 of the 598 tested for SARS-CoV-2.

### 2.2. Data Analysis

In the dataset provided, we have divided the extracted information into columns and rows. Rows are referred to as observations. Each column in this dataset shows some information about observations, such as hematocrit, hemoglobin, or platelets. These columns are labeled features or predictor variables of our dataset. The “SARS-CoV-2 exam result” column classifies our dataset and predicts whether or not the individual is infected with COVID-19; consequently, it is considered as the target variable.

The degree of influence that the variables in the dataset have over the target value can be determined by their correlation with the target. As a result, we were able to pinpoint the features that can distinguish an infected patient from a noninfected patient.

**Figure 1:**
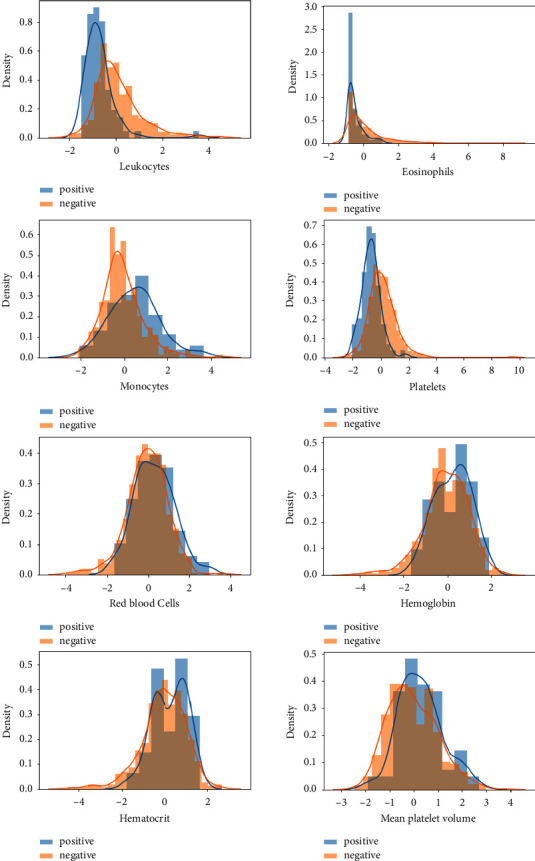
Visualization of blood/target. The plots show the variation curves of individual parameters categorized according to whether the patient tested positive (blue curve) or negative (yellow curve) on the RT-PCR test for SARS-CoV-2. These plots indicate a statistically significant difference between the two curves (positive-negative). In particular, leukocytes, eosinophils, monocytes, and platelets seem to have different variability across the two classes (negative-positive).

**Figure 2:**
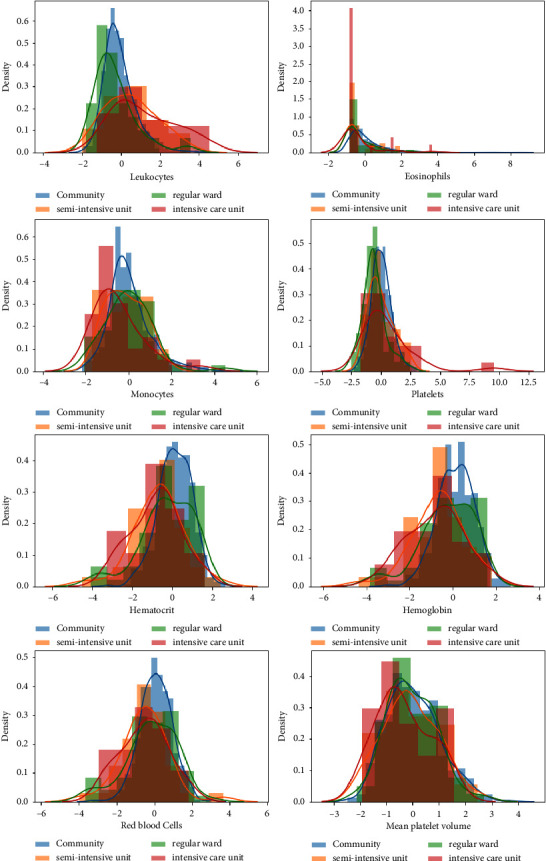
Visualization of hospitalization/blood. Monocytes, eosinophils, leukocytes, and platelets seem to have different variability between COVID-19-positive and negative patients. In addition, the levels of these parameters vary according to patients' hospitalization status.

During our data mining and analysis of the “blood/target and hospitalization/blood” visualization graphs, we noticed that the monocyte, platelet, leukocyte, and eosinophil levels for infected and noninfected individuals were significantly different (see Figure 1). In addition, the relationship between a patient's hospitalization status and their blood characteristics differed for each hospitalization category (community, regular ward, semi-intensive unit, or intensive care unit) (see Figure 2), which presents the possibility that these variables are related to positive COVID-19 infection. Testing this hypothesis through Student's *t*-test allowed us to verify that the means (averages) between the two distributions (positive versus negative COVID-19 test result) are significantly different at the level of these variables.

Student's *t*-test results (see Table 3) support our hypothesis that the levels of platelets, monocytes, eosinophils, and leukocytes are significant for predicting SARS-CoV-2 and, therefore, can assist in decision-making.

### 2.3. Data Preprocessing

Data preprocessing consists of treating, fixing, and preparing data before inputting it for machine learning. The goal is to transform the raw data into a format conducive for the development of a machine-learning model and to clean the dataset as much as possible to improve the performance of the model. For our data, we followed a simple and efficient approach. The dataset consists of columns with continuous and categorical variables. Since the machine-learning model requires that all input data be in numeric form, we have coded the target value “SARS-CoV-2 exam result” by assigning 0 for “negative” and 1 for “positive.”

The hospital data contain 111 columns with 90% missing values (5,046 of the 5,644 results). The dataset is also challenging because no information is provided about the patients except their ages, which makes it difficult to fill out the missing data using precise extrapolation methods. Using different methods to recover the missing data using the mean value is effective for some cases, but not for a set of medical exam results (sensitive data). For all these reasons, 5,046 of the 5,644 results were excluded from analysis, leaving only 598 cases (517 positives and 81 negatives) containing complete variables for use in the study.

The analysis was performed based on patients' severity according to their hospitalization status. Blood counts were obtained for the community, regular ward, semi-intensive unit, and intensive care unit cohorts (see Table 2). Only patients with a full blood examination and RT-PCR SARS-CoV-2 outcome were included.

To ensure that our prediction is based on early indicators, patients in the semi-intensive and intensive care units were removed from our analysis. In addition, we excluded pathogenic (viral) factors and age from our study.

In this work, we used feature selection using the SelectKbest transformer and polynomial features in both groups of the dataset to find the most important variables. Given the result of our statistical test (Table 3), we will examine only the blood variables. These variables will be used to detect the presence of SARS-CoV-2.

### 2.4. Evaluation Metrics

The purpose of this study was to accurately predict whether an individual is infected with COVID-19 based on available clinical data. The main issue in this study is the unbalanced classes. Since this is a very sensitive prediction, accuracy alone is typically not sufficient in the absence of other performance measures. In this case, we used a confusion matrix to evaluate the performance of classification models. Four indicators are measured in the confusion matrix: accuracy, recall, precision, and *F*1 score (see Table 4) [25]. These indicators are defined as follows.

The terms used in the equations are *a*, true positive; *d*, true negative; *b*, false positive; *c*, false negative; *r*, recall; and *p*, precision.

#### 2.4.1. Accuracy

Accuracy is the percentage of all predictions that were accurate.

The formula is

(1) $$\begin{array}{c} Accuracy=\frac{a+b}{a+b+c+d}\text{.} \end{array}$$

#### 2.4.2. Precision (Positive Predictive Value)

Precision is the likelihood that the prediction of a positive result is actually positive. Precision minimizes the chances of a false positive result.

The formula is

(2) $$\begin{array}{c} p=\frac{a}{a+b}\text{.} \end{array}$$

#### 2.4.3. Recall (True Positive Rate, Sensitivity, or Probability of Detection)

Recall is the probability of the model successfully identifying true positive cases. The recall reduces the number of false negatives in our predictions, allowing us to detect as many COVID-19-infected individuals as possible.

The formula is

(3) $$\begin{array}{c} r=\frac{a}{a+c}\text{.} \end{array}$$

#### 2.4.4. *F*1 Score

The *F*1 score is an overall indicator of classifier performance, and *F*1 is a function of a true positive rate and positive predictive value (precision and recall).

The formula is

(4) $$\begin{array}{c} F1_{Score}=\frac{2*p*r}{p+r}\text{.} \end{array}$$

#### 2.4.5. AUC

AUC is the area beneath the ROC curve. It is calculated using the ROC curve, which is a plot of the true positive rate versus the false positive rate. The greater the area under the plotted line, the better the algorithm performs due to its higher sensitivity and specificity. The commonly used metric known as the “area under the ROC curve,” or “AUROC,” offers an easy approach to compare algorithms. Many investigators choose the point on the ROC curve that, in their opinion, will produce the best outcomes for the task [26].

### 2.5. Methods

The purpose of this work was to predict COVID-19 infections from blood features using SVM and KNN classifiers. After preprocessing and feature selection, we applied the two models to the bloodwork results for patients with and without COVID-19 who either were not hospitalized (39 tested positive for SARS-CoV-2 and 431 negative) or were admitted to the regular ward (26 tested positive for SARS-CoV-2 and 31 negative). A common supervised-learning technique used in regression and classification is SVM [27]. SVM involves finding a hyperplan, whose ideal location is in the center of two classes. The best hyperplan equation is that which maximizes the margin between the two groups in various classes [28]. The choice of the kernel function is an essential component because a suitable kernel function is imperative for the SVM to acquire learning capability. Therefore, we employ SVM with the radial basis kernel function.

Meanwhile, the KNN algorithm is considered a type of lazy learning since it is practical machine learning that does not require preparation or a training cycle. Because of its straightforwardness, the KNN calculation is one of the ten best known data-mining algorithms [29]. KNN demonstrates high proficiency and a magnificent capacity to tackle troublesome classification problems. As a rule, KNN is a valuable and quick procedure [30], which lends itself to our purpose of saving valuable time for health experts.

**Figure 3:**
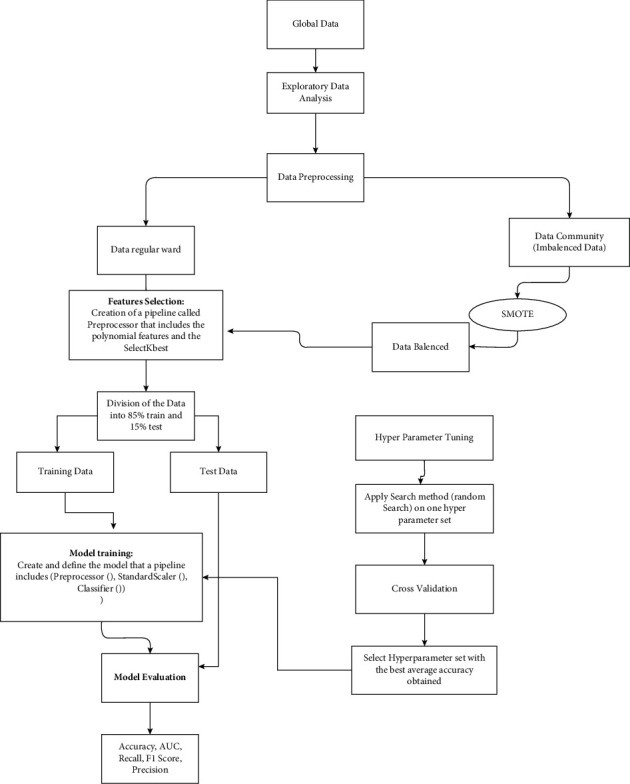
Methodology workflow.

Therefore, we implemented and regularized the two models as follows (see Figure 3). First, we created a list of models that included SVM and KNN and then submitted all the models to the same evaluation procedure. We note that the algorithms in the list are introduced through a pipeline that includes steps completed in the preprocessing phase. Then, we renamed this pipeline that has the polynomial features and SelectKbest transformers as “preprocessors.”

This preprocessor pipeline is appended, upstream, to these two models. In contrast, we created a new pipeline of the SVM model that contains the preprocessor followed by a standardization operation (with the StandardScaler function) and an SVC classifier. In addition, we applied the same process to KNN, which is a pipeline containing the preprocessor, StandardScaler, and KNeighborsClassifier. We trained and evaluated both models on their default hyperparameters using our evaluation procedure. (The evaluation function provides training and testing of the models, as well as visualization of the confusion matrix and the learning curve.) Our goal was to improve the performance of these models by enhancing these hyperparameters.

#### 2.5.1. Hyperparameter-Tuning Techniques

The random search technique via the RandomizedSearchCV function enables identification of the best hyperparameters by comparing the performance of each combination using the cross-validation technique. We created a dictionary containing the different hyperparameters (penalty coefficient C, Gamma, polynomial feature, and SelectKbest) to be regulated. We embedded the SVM model, which is a pipeline, as well as the dictionary of hyperparameters in the function RandomizedSearchCV, followed by a scoring rubric which is the recall with cross-validation (cv = 10). We applied the same process to KNN using the RandomizedSearchCV function. It included the KNN model, a dictionary of hyperparameters (“neighbors classifier weights,” “neighbors classifier neighbors,” and “polynomial features degree,” SelectKbest k), followed by a scoring rubric, which is always the recall with cross-validation equal to 10 and the number of iterations fixed at 100.

#### 2.5.2. Application of SMOTE to the Imbalanced Dataset (Community)

The community dataset included 39 positive and 431 negative patients. Therefore, the data are characterized by a distribution of the modalities of the class that is very far from a uniform distribution (that is, unbalanced classes), which is a relatively frequent situation in some classifications. More concretely, unbalanced classes generally refer to a classification problem where the classes are not equally distributed. The difficulty of working with unbalanced data classes (defined as positive/negative = 0.09) is that the KNN and SVM models ignore the minority class. A class imbalance increases the difficulty of learning via the classification algorithm. Indeed, the algorithm has few examples of the minority class to learn from. It is therefore biased and produces potentially less robust predictions than if the data were balanced.

The imbalance between the two classes in the community dataset is significant (positive/negative = 0.09), thereby degrading the performance of the defined ML model. Thus, the SMOTE technique [31] is adapted to balance the two classes in the dataset, a type of data augmentation for the minority class, 1, and designed to make it similar to the majority class, 0. We used the implementations of SMOTE provided by the Python library imbalanced-learn set to their default parameters (k neighbors = 5 …); this object is an implementation of [31]. The 10-fold stratified cross-validation technique is again applied and repeated recursively for 10 classes.

Next, we divided the dataset, designating 85% of the data points for training and 15% for testing. Finally, we implemented and evaluated the SVM and KNN models, as shown in Figure 3.

## 3. Results

In this section, the effectiveness of the proposed SARS-CoV-2 prediction strategy is evaluated. The proposed SVM and KNN classifiers will be evaluated for both regular ward and community groups to accurately detect SARS-CoV-2 patients. The performance of each implemented model is presented in terms of AUC, accuracy, precision, recall, and *F*1 score.

### 3.1. Statistical Analysis

Polynomial features and SelectKbest provide more information about the most important variables. SelectKbest selects the 14 variables with a statistical test score of dependence (ANOVA test) with the target. These variables are the most significant for predictive purposes. The dependency test analysis of the main variables according to the SelectKbest transformer corresponding to the patients in the regular ward (see Table 5) and the community ward revealed a recognizable ANOVA test score. It should be noted that the four variables from the regular ward data had the best scores for eosinophils, followed by red blood cells, hemoglobin, and leukocytes (see Table 6). Community patients with SARS-CoV-2 have high scores on leukocyte, monocyte, platelet, and eosinophil parameters.

### 3.2. Results for Patients Admitted to the Regular Ward

**Figure 4:**
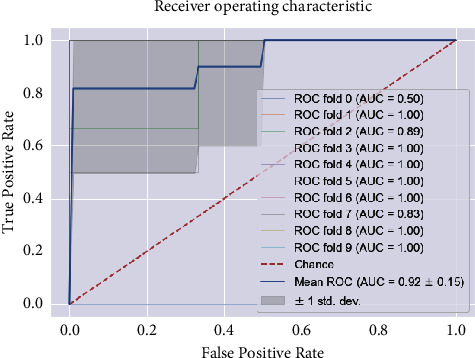
The ROC curve and AUC values of the SVM model for regular ward patients. The ROC curve shows the true-positive rate versus the false-positive rate. Comparing AUC values reveals that the ROC curve has greater AUC and thus indicates better overall performance. Generally, the higher the AUC, the better the model performance.

**Figure 5:**
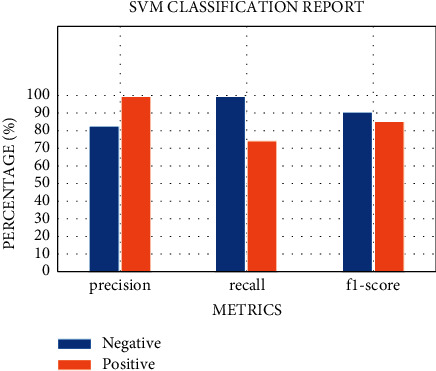
The metrics of the SVM model for regular ward patients.

**Figure 6:**
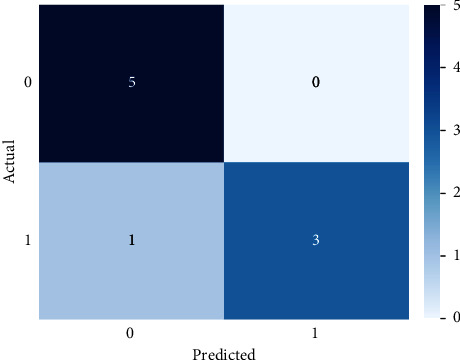
Confusion matrix of the SVM model for regular ward patients.

**Figure 7:**
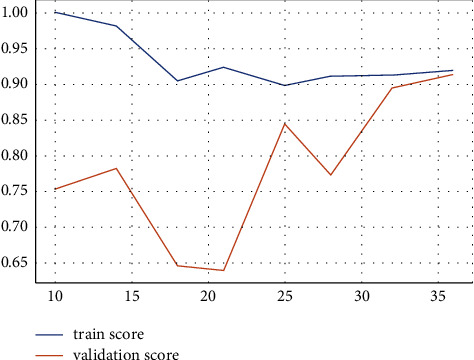
Learning and validation curve of the SVM model for regular ward patients.

SVM, run using the default settings, yields a precision value of 89% and a recall value of 75% and a precision and *F*1 score of 1 and 86%, respectively, for class 1 (patients testing positive) on 10-fold stratified cross-validation, as shown in Table 7. Receiver operating characteristic (ROC) curves were plotted for the 10-fold and area under the curve (ROC) values for all folds (Figure 4). After the model was improved through optimization of its parameters via a random search, we obtained almost the same values for the model metrics. The metric evaluations of the model are presented in Figure 5. The confusion matrix and the learning and validation curve are illustrated in Figures 6 and 7.

**Figure 8:**
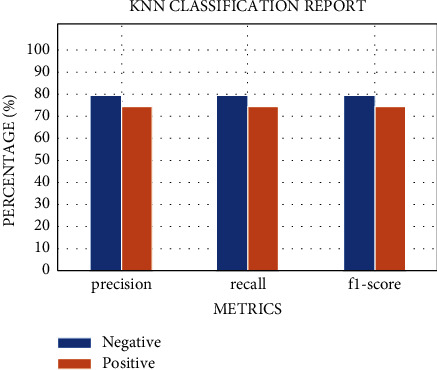
The metric evaluations of the KNN model for regular ward patients.

**Figure 9:**
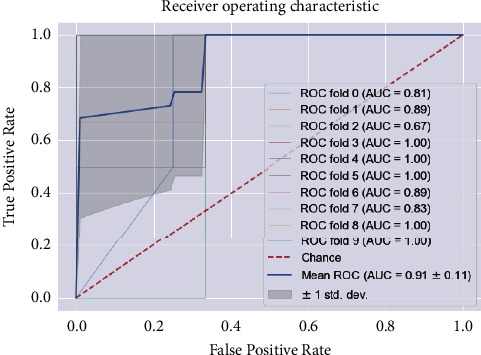
The ROC curve and AUC values of the KNN model for regular ward patients.

The results of the implementation of KNN with default parameters yield an average AUC of 84% on 10-fold stratified cross-validation, an accuracy of 78%, a recall of 75%, a precision of 75%, and an *F*1 score of 75%, respectively, for the class 1 patients. After regularization of the hyperparameters, the AUC improved remarkably, from 84% to 91%. While the other metrics remain almost the same (Figure 8), the AUC score is equal to 0.91 ± 0.11 (see Figure 9). The results of the two classifiers are summarized in Tables 7 and 8.

### 3.3. Implementation Results for the Community Dataset

**Figure 10:**
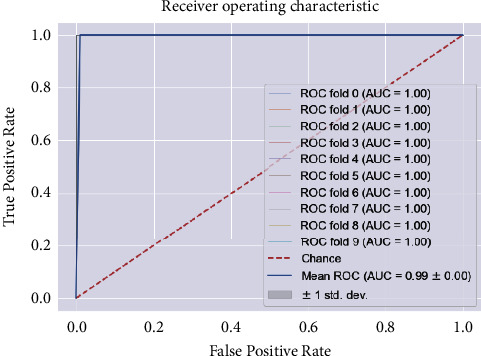
The ROC curve and AUC values of the SVM model for community patients. Comparing AUC values for algorithm simulation cases (Figures 6 and 7) shows that the ROC curve for the SVM model with community patients has greater AUC and, thus, indicates better model performance.

**Figure 11:**
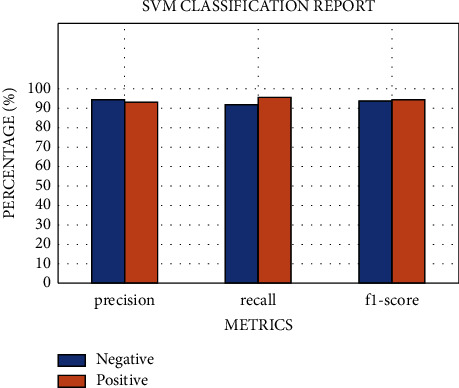
The metrics of the SVM model for community patients.

**Figure 12:**
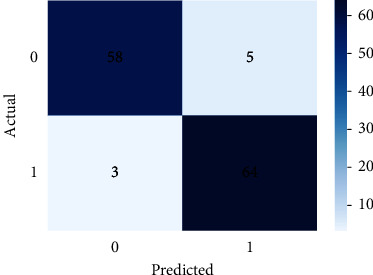
Confusion matrix of the SVM model for community patients.

**Figure 13:**
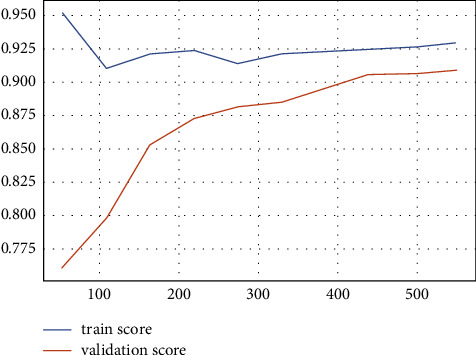
Learning and validation curves of the SVM model for community patients.

SVM defined for the data on patients not admitted to hospital using default parameters yields an average accuracy of 85%, a recall of 81%, a precision of 89%, an *F*1 score of 84%, and an AUC of 99% for class 1 on 10-fold stratified cross-validation (see Table 9). The ROC curves for all 10-folds produce an average AUC of 0.99 ± 0.00. Meanwhile, the optimized SVM results yield an average AUC of 99% (see Figure 10), an accuracy of 95%, a recall of 96%, a precision of 94%, and an *F*1 score of 95%, respectively (see Table 10 and Figure 11). The confusion matrix, learning curve, and validation curve are illustrated in Figures 12 and 13.

With KNN set to default parameters, accuracy is 88%, recall is 96%, precision is 84%, and the *F*1 score is 90%, respectively (see Table 9).

**Figure 14:**
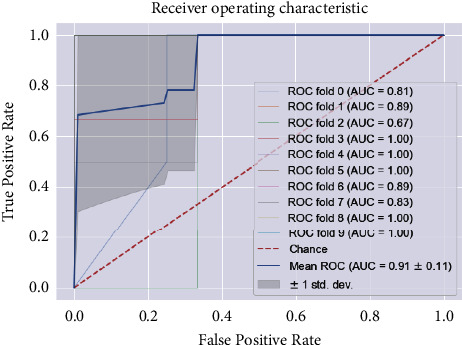
The ROC curve and AUC values of the KNN model for community patients.

**Figure 15:**
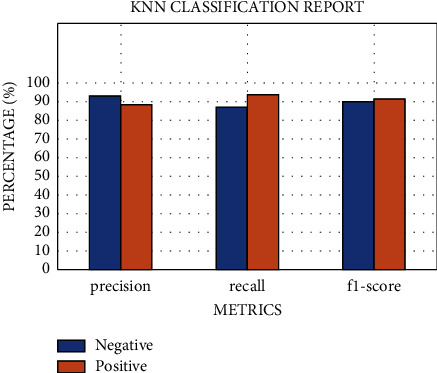
The metric evaluations of the KNN model for community patients.

After tuning parameters and 10-fold stratified cross-validation are applied, the mean AUC score is 0.99 ± 0.10 (see Figure 14), accuracy is 90%, recall is 91%, precision is 89%, and the *F*1 score is 91%, respectively (see Table 10 and Figure 15). The results of the two classifiers are summarized in Tables 9 and 10.

## 4. Discussion

The complete dataset included 5,644 patients tested between March 28, 2020, and April 3, 2020, of which 598 complete blood count results were used for statistical analysis. The remaining 5,046 results were omitted because of incomplete blood count data. Despite the constraint of the small sample size, our goal of identifying patients with COVID-19 infection was achieved with an accuracy of 95% using an SVM classifier. By promoting and developing different classification models (SVM and KNN) with 598 patients, we predicted SARS-CoV-2 infection with an AUC of up to 0.99 for nonhospitalized patients and 0.92 for regular ward patients, using only standardized complete blood count data.

The finding that SARS-CoV-2-positive and negative cases can be classified using biological features at an early stage of the disease has important implications. The study was performed on a dataset organized by the patients' hospitalization status. We excluded patients in the semi-intensive and intensive care units from our analysis in order to base predictions of COVID-19 test results on indicators of the disease in its early phase. Therefore, age and pathogenic (viral) variables were excluded from our study.

The strategy we have developed can provide a reliable and rapid SARS-CoV-2 diagnosis. KNN and SVM algorithms on both groups of patient data have shown that the SVM algorithm applied to community patients with optimization and the SMOTE technique offers the most accurate predictions. This enhanced SVM technique provides precision, recall, accuracy, and AUC values that reach 0.94, 0.96, 0.95, and 0.99, respectively. KNN optimized over community patient data after the SMOTE technique is applied produces accurate results.

However, for the regular ward data, both classifiers retain almost identical metrics regardless of the optimization of the hyperparameters and the selection technique used. This result may be due to the problem of low data registration, which influences and explains both the lack of improvement in the results despite optimization. The same problems may also influence and provide less relevant information for the significant variables of the regular ward patients as well (see scoring table). Overall, this underscores the difficulty of interpreting standardized data of low registration. Both classifiers can be used as an improved algorithm to perform SARS-CoV-2 prediction for new data. SVM and KNN are very robust in analyzing data with two classes (positive or negative).

The symptoms of COVID-19 are often accompanied by an immune response [32]. Therefore, hyperactivity of blood parameters is noticed in all stages whenever an infection exists [33]. Indeed, several scientific reports have confirmed this hypothesis. The researchers in these works used similar predictive models based on blood parameters, suggesting elevated levels of some of these parameters; for example, an elevated level of eosinophilia could be a potential diagnostic indicator [34]. Indeed, the value of this marker has been identified in cerebrovascular pathologies and during coronary bypass surgery. The previous finding of an elevated neutrophil/lymphocyte count seems to be a relevant marker in the diagnosis of COVID-19 [35], as is the case in our study (see the tables of the predictive variables for each dataset).

In this study, we examined the evolution of blood parameters in all the patients in a particular unit as exploratory analyses using ratios of diagnostic blood characteristics. However, the fact that these characteristics may be related to other pathogens and viral diseases is a potential limitation of the proposed method. Indeed, previous studies have shown that MERS increases monocytes [36]. SARS also directly infects monocytes, which produce cytokines that directly affect neutrophils [37]. Both infections, then, produce similar effects on blood activity related to humanitarian reactions. At the same time, this study indicates the immediate relation of the pathogenesis of COVID-19 to monocytes and neutrophils as shown in the dependency test score results (ANOVA test) (see Tables 5 and 6).

However, these parameters are often significant depending on the results of the statistical test performed, and we have initiated research to identify feature scores that distinguish SARS-CoV-2 with a preprocessor that embraces both polynomial features and the SelectKbest transformer in both groups of the dataset to find the most significant variables (see Score Table). The variable selection method used shows, on the one hand, the utility of data mining in extracting altered information from key features for classification should a future strain of coronavirus emerge, which remains a risk and danger facing humanity. On the other hand, the collection, analysis, and processing of medical data must be of interest to the health sector.

## 5. Conclusion

Our model and all artificial intelligence-based predictive models related to the healthcare sector rely on medical data. The use of machine learning (ML) is important for processing patient data to guide effective control and treatment strategies for the pandemic. The main element in constructing an AI-based predictive model is information. Therefore, the availability of and access to such data are crucial for the development of similar studies. The study will also be further adapted to address the lack of information and collected data in the medical field to facilitate the task of direct detection of SARS-CoV-2 in hospitals and medical testing laboratories. An automated medical diagnosis that reduces costs for healthcare institutions is very important, especially when quick decisions are necessary to isolate infected patients and provide prompt treatment. Direct contact with infected patients may threaten doctors and caregivers with illness or even death. To overcome this global and dangerous challenge, it is fundamentally essential to analyze patient data at health facilities and detect the disease immediately, with accuracy, and within the shortest possible time frame. Future work will focus on creating a pipeline that combines AI-based predictive models with these types of complete blood counts and healthcare data processing models. These models will then be included in applications that will help in the development of mobile healthcare. Therefore, ML can provide a step toward a semiautonomous, expeditious diagnostic system that would be useful in combating a future pandemic situation and would offer tremendous opportunities to harmonize with sustainable development goals.

## Figures and Tables

**Table 1:** Data description.

Features	Description
Target	SARS-CoV-2 exam result (RT-PCR SARS-CoV-2 test)

Id	Patient ID

Age	Patient age quantile

Categorical variables	Pathogen test

Continuous variables	Complete blood count

Missing values	90% NaN

Hospitalization	Community
	Regular ward
	Semi intensive unit
	Intensive care unit

**Table 2:** Albert Einstein hospital dataset groups.

Group of patients	RT-PCR negative	RT-PCR positive	Other pathogens
Community	431 (92%)	39 (8%)	149 (32%)
Regular ward	31 (54%)	26 (46%)	12 (21%)
Semi-intensive unit (SIU)	34 (81%)	8 (19%)	17 (40%)
Intensive care unit (ICU)	21 (72%)	8 (28%)	12 (41%)
Total	517 (86%)	81 (14%)	190 (31%)

**Table 3:** Student *t*-test results.

Parameters	*p* value	*H*_0_
Hematocrit	*p* > 0.05	0
Hemoglobin	*p* > 0.05	0
Platelets	*p* < 0.05	*H*_0_ rejected
Mean platelet volume	*p* < 0.05	*H*_0_ rejected
Red blood cells	*p* > 0.05	0
Lymphocytes	*p* > 0.05	0
Mean corpuscular hemoglobin concentration (MCHC)	*p* > 0.05	0
Leukocytes	*p* < 0.05	*H*_0_ rejected
Basophils	*p* > 0.05	0
Mean corpuscular hemoglobin (MCH)	*p* > 0.05	0
Eosinophils	*p* < 0.05	*H*_0_ rejected
Mean corpuscular volume (MCV)	*p* > 0.05	0
Monocytes	*p* < 0.05	*H*_0_ rejected
Red blood cell distribution width (RDW)	*p* > 0.05	0

The null hypothesis (*H*_0_) stated that both means are statistically equal, whereas alternative hypothesis stated that both means are not statistically equal.

**Table 4:** Performance metrics.

Confusion matrix		Target		
		Positive	Negative	
Model	Positive	a	b	Positive predictive value = *a*/(*a *+* b*)
	Negative	c	d	Negative predictive value = *d*/(*c* + *d*)

**Table 5:** The 14 variables (14 different blood counts) in the construction of the predictive model (regular ward data), ranked according to the best dependency test scores (ANOVA test) with the target (SARS-CoV-2).

Feature name	Score
Eosinophils	19.097924
Red blood cells	10.508018
Hemoglobin	9.878300
Leukocytes	9.760907
Hematocrit	9.638821
Mean platelet volume	5.262199
Platelets	4.385559
Red blood cell distribution width (RDW)	3.503271
Basophils	3.459295
Lymphocytes	2.726842
Mean corpuscular volume (MCV)	1.767816
Mean corpuscular hemoglobin (MCH)	1.447305
Monocytes	0.137198
Mean corpuscular hemoglobin concentration (MCHC)	0.008929

**Table 6:** The 14 variables (14 different blood counts) in the construction of the predictive model (community data), ranked according to the best dependency test scores (ANOVA test) with the target (SARS-CoV-2).

Feature name	Score
Leukocytes	375.774996
Monocytes	228.273420
Platelets	225.528765
Eosinophils	46.271648
Mean platelet volume	34.246066
Mean corpuscular hemoglobin (MCH)	12.148960
Hemoglobin	10.499801
Red blood cell distribution width (RDW)	10.174787
Basophils	8.583406
Hematocrit	7.795479
Mean corpuscular volume (MCV)	6.610441
Lymphocytes	6.433391
Mean corpuscular hemoglobin concentration (MCHC)	6.345696
Red blood cells	1.443602

**Table 7:** Evaluation results for model predictions with the regular ward data group (patients testing positive for SARS-CoV-2) using default parameters.

Variables	Model	Precision	Recall	*F*1 score	Accuracy	AUC
14 different blood counts	SVM	1.00	0.75	0.86	0.89	(0.94 ± 0.15)
14 different blood counts	KNN	0.75	0.75	0.75	0.78	(0.84 ± 0.20)

**Table 8:** Evaluation results for model predictions with the regular ward data group (patients testing positive for SARS-CoV-2) after tuning parameters.

Variables	Model	Precision	Recall	*F*1 score	Accuracy	AUC
14 different blood counts	SVM	1.00	0.75	0.86	0.89	(0.92 ± 0.15)
14 different blood counts	KNN	0.75	0.75	0.75	0.78	(0.91 ± 0.11)

**Table 9:** Evaluation results for model predictions with the community data group (patients testing positive for SARS-CoV-2) using default parameters.

Variables	Model	Precision	Recall	*F*1 score	Accuracy	AUC
14 different blood counts	SVM	0.89	0.81	0.84	0.85	(0.99 ± 0.00)
14 different blood counts	KNN	0.84	0.96	0.90	0.88	(0.99 ± 0.00)

**Table 10:** Evaluation results for model predictions with the community data group (patients testing positive for SARS-CoV-2) after tuning parameters.

Variables	Model	Precision	Recall	*F*1 score	Accuracy	AUC
14 different blood counts	SVM	0.94	0.96	0.95	0.95	(0.99 ± 0.00)
14 different blood counts	KNN	0.89	0.91	0.91	0.90	(0.99 ± 0.11)

## Data Availability

The data used to support the findings of this study are available at https://www.kaggle.com/einsteindata4u/covid19.

## References

[1] World Health Organization (WHO) (2020). Virtual press conference on COVID-19. https://www.who.int/docs/default-source/coronaviruse/transcripts/who-audio-emergencies-coronavirus-press-conference-full-and-final-11mar2020.pdf.

[2] Worldometers (2020). https://www.worldometers.info/coronavirus/.

[3] World Health Organization (WHO) (2020). WHO covid-19 strategy update. https://www.who.int/docs/default-source/coronaviruse/covid-strategy-update-14april2020.pdf.

[4] Hasan I., Dhawan P., Rizvi S. A. M., Dhir S. (2022). Data analytics and knowledge management approach for COVID-19 prediction and control. *International Journal on Information Technology*.

[5] Iwendi C., Bashir A. K., Peshkar A. (2020). COVID-19 patient health prediction using boosted random forest algorithm. *Frontiers in Public Health*.

[6] Thamilselvana P., Sathiaseelan J. (2015). A comparative study of data mining algorithms for image classification. *International Journal of Education and Management Engineering*.

[7] Kharya S., Soni S., Swarnkar T. (2022). Generation of synthetic datasets using weighted bayesian association rules in clinical world. *International Journal on Information Technology*.

[8] Sethy P. K., Behera S. K. (2020). Detection of coronavirus disease (covid-19) based on deep features. *Electrical and Electronic Engineering*.

[9] Gayathri G. V., Satapathy S. C. (2020). A Survey on techniques for prediction of asthma. *Smart Intelligent Computing and Applications: Proceedings of the Third International Conference on Smart Computing and Informatics*.

[10] Ribeiro M. H. D. M., da Silva R. G., Mariani V. C., Coelho L. S. (2020). Short-term forecasting COVID-19 cumulative confirmed cases: perspectives for Brazil. *Chaos, Solitons & Fractals*.

[11] Da Silva R. G., Ribeiro M. H. D. M., Mariani V. C., Coelho L. S. (2020). Forecasting Brazilian and American COVID-19 cases based on artificial intelligence coupled with climatic exogenous variables. *Chaos, Solitons and Fractals*.

[12] Yao H., Zhang N., Zhang R. (2020). Severity detection for the coronavirus disease 2019 (COVID-19) patients using a machine learning model based on the blood and urine tests. *Frontiers in Cell and Developmental Biology*.

[13] Mahmud T., Rahman M. A., Fattah S. A. (2020). CovXNet: a multi-dilation convolutional neural network for automatic COVID-19 and other pneumonia detection from chest X-ray images with transferable multi-receptive feature optimization. *Computers in Biology and Medicine*.

[14] Keshinro S. O., Awolola N. A., Adebayo L. A., Mutiu W. B., Saka B. A., Abdus-Salam I. A. (2021). Full autopsy in a confirmed COVID-19 patient in Lagos, Nigeria–A case report. *Human Pathology: Case Reports*.

[15] Ribeiro M. H. D. M., da Silva R. G., Larcher J. H. K., Mariani V. C., Coelho L. D. S. (2022). Ensemble learning models coupled with urban mobility information applied to predict COVID-19 incidence cases. *Modeling, Control and Drug Development for COVID-19 Outbreak Prevention*.

[16] Altantawy D. A., Kishk S. S. (2023). Equilibrium-based COVID-19 diagnosis from routine blood tests: a sparse deep convolutional model. *Expert Systems with Applications*.

[17] Beck B. R., Shin B., Choi Y., Park S., Kang K. (2020). Predicting commercially available antiviral drugs that may act on the novel coronavirus (SARS-CoV-2) through a drug-target interaction deep learning model. *Computational and Structural Biotechnology Journal*.

[18] Khalifa N. E. M., Taha M. H. N., Hassanien A. E., Elghamrawy S. (2022). Detection of coronavirus (COVID-19) associated pneumonia based on generative adversarial networks and a fine-tuned deep transfer learning model using chest X-ray dataset. *Proceedings of the 8th International Conference on Advanced Intelligent Systems and Informatics*.

[19] Gunraj H., Wang L., Wong A. (2020). Covidnet-ct: a tailored deep convolutional neural network design for detection of covid-19 cases from chest ct images. *Frontiers of Medicine*.

[20] Aggarwal P., Mishra N. K., Fatimah B., Singh P., Gupta A., Joshi S. D. (2022). COVID-19 image classification using deep learning: advances, challenges and opportunities. *Computers in Biology and Medicine*.

[21] Ahuja S., Panigrahi B. K., Dey N., Taneja A., Gandhi T. K. (2022). McS-Net: multi-class Siamese network for severity of COVID-19 infection classification from lung CT scan slices. *Applied Soft Computing*.

[22] Jyoti K., Sushma S., Yadav S., Kumar P., Pachori R. B., Mukherjee S. (2023). Automatic diagnosis of COVID-19 with MCA-inspired TQWT-based classification of chest X-ray images. *Computers in Biology and Medicine*.

[23] Paulo S. (2020). Brazil: diagnosis of Covid-19 and its clinical spectrum. https://www.kaggle.com/einsteindata4u/covid19.

[24] CoronaHack (2020). AI vs covid-19. https://www.coronahack.co.uk/.

[25] Prasetyo E. (2019). *Data Mining Mengolah Data Menjadi Informasi Menggunakan Matlab*.

[26] Handelman G. S., Kok H. K., Chandra R. V. (2019). Peering into the black box of artificial intelligence: evaluation metrics of machine learning methods. *American Journal of Roentgenology*.

[27] Huang S., Cai N., Pacheco P. P., Narrandes S., Wang Y., Xu W. (2018). Applications of support vector machine (SVM) learning in cancer genomics. *Cancer genomics & proteomics*.

[28] Pratama M. O., Satyawan W., Jannati R. (2019). The sentiment analysis of Indonesia commuter line using machine learning based on twitter data Journal of Physics: conference Series. *Journal of Physics: Conference Series*.

[29] Zhang S., Li X., Zong M., Zhu X., Wang R. (2018). Efficient kNN classification with different numbers of nearest neighbors. *IEEE Transactions on Neural Networks and Learning Systems*.

[30] Jaafor O., Birregah B. (2020). KNN-LC: classification in unbalanced datasets using a KNN-based algorithm and local centralities. *Data-Driven Modeling for Sustainable Engineering: Proceedings of the First International Conference on Engineering, Applied Sciences and System Modeling (ICEASSM)*.

[31] Chawla N. V., Bowyer K. W., Hall L. O., Kegelmeyer W. P. (2002). SMOTE: synthetic minority over-sampling technique. *Journal of Artificial Intelligence Research*.

[32] Qin C., Zhou L., Hu Z. (2020). Dysregulation of immune response in patients with coronavirus 2019 (COVID-19) in Wuhan, China. *Clinical Infectious Diseases*.

[33] Yang X., Yang Q., Wang Y. (2020). Thrombocytopenia and its association with mortality in patients with COVID-19. *Journal of Thrombosis and Haemostasis*.

[34] Zhang J. J., Dong X., Cao Y. Y. (2020). Clinical characteristics of 140 patients infected with SARS-CoV-2 in Wuhan, China. *Allergy*.

[35] Yang A. P., Liu J. P., Tao W. Q., Li H. M. (2020). The diagnostic and predictive role of NLR, d-NLR and PLR in COVID-19 patients. *International Immunopharmacology*.

[36] Min C. K., Cheon S., Ha N. Y. (2016). Comparative and kinetic analysis of viral shedding and immunological responses in MERS patients representing a broad spectrum of disease severity. *Scientific Reports*.

[37] Hu W. J., Yen Y. T., Singh S., Kao C. L., Wu-Hsieh B. A. (2011). SARS-CoV regulates immune function-related gene expressions in human monocytic cells. *Nature Precedings*.

